# Adhesion Behavior of *Escherichia coli* Strains on Glass: Role of Cell Surface Qualitative and Quantitative Hydrophobicity in Their Attachment Ability

**DOI:** 10.1155/2021/5580274

**Published:** 2021-10-07

**Authors:** Kaoutar Elfazazi, Hafida Zahir, Safae Tankiouine, Btissam Mayoussi, Chorouk Zanane, Souad Lekchiri, Mostafa Ellouali, El Mostafa Mliji, Hassan Latrache

**Affiliations:** ^1^Laboratory of Bioprocess and Bio-Interfaces, Faculty of Sciences and Technics, Beni Mellal, Morocco; ^2^Agro-Food Technology and Quality Laboratory, Regional Center of Agricultural Research of Tadla, National Institute of Agricultural Research, Avenue Ennasr, BP 415 Rabat Principale, 10090 Rabat, Morocco; ^3^Department of Environment and Food Safety, Pasteur Institute, Casablanca, Morocco

## Abstract

Microbial adhesion to surfaces is thought to involve physicochemical interactions between the substrate and microbial cells. Understanding the physicochemical aspects involved in the adhesion phenomenon, as a critical step in biofilm formation, is essential to finding ways to prevent their formation and control biocontamination risks. The aim of this study was to investigate the relation between the adhesion behavior of 12 *Escherichia coli* strains isolated from food and their surface hydrophobicities using qualitative (*θ*_*w*_) and quantitative (Δ*G*_*iwi*_) approaches. The surface physicochemical properties of both bacterial cells and glass material were estimated through contact angle measurements. The adhesive behavior of *E. coli* strains on a glass surface was assessed. The results showed a good logarithmic relation between the percentage of the adhered cells and their surface hydrophobicity with the quantitative approach Δ*G*_*iwi*_; however, qualitative hydrophobicity (*θ*_*w*_) appeared to demonstrate no effect regarding adhesion behavior. This work lays the foundation for future studies and opens an important debate on the mechanisms underlying the adhesion behavior of *E. coli* strains by using the thermodynamic approach (Δ*G*_*iwi*_) as an important model of hydrophobicity that could explain and predict better bacterial adhesion ability.

## 1. Introduction

In natural environments, as well as in the food industry, microorganisms are most often attached to solid surfaces and are generally provided with sufficient nutrients to ensure their viability and growth. This adhesion ability of bacteria to surfaces is cause for concern for many industries, particularly the food industry. In fact, microbial adhesion to surfaces can act as a source of contamination, which may compromise hygienic and food quality [1]. *E. coli* is an important foodborne pathogen that causes thousands of cases of foodborne diseases annually across the world [2, 3]. Several studies have been conducted, therefore, to determine the factors and mechanisms leading to the microbial attachment to food contact surfaces [4–10].

Microbial adhesion is a complex phenomenon involving several parameters and energy interactions, among which the most remarkable are the physicochemical properties of bacterial and support surfaces [4, 7–14]. The interactions involved in this process are mainly Lifshitz–van der Waals, electrostatic [15], and Lewis (acid-base) ones [16]. These physicochemical interactions depend on the physicochemical properties of both the substratum and bacterial surface including hydrophobicity [4, 9, 15, 17] and electron donor-electron acceptor (acid-base) characteristics [10, 18].

Bacterial hydrophobicity generally refers to the tendency of a bacterial cell to interact with cells of similar hydrophobicity as opposed to water (*θ*_*w*_) [19]. Most previous works have used the qualitative hydrophobicity (*θ*_*w*_) measured directly by contact angle with water to explain the adhesion behavior of many bacterial species [15, 20–22]. However, several studies reported that the qualitative hydrophobicity expressed as wettability with water (*θ*_*w*_) could not completely explain the adhesion behavior of many bacteria [4, 7, 20, 21, 23]. Therefore, the free energy of interaction (Δ*G*_*iwi*_) was proposed by Van Oss et al. [24] as a quantitative method to assess cell hydrophobicity. This parameter is directly related to the interfacial tension and considers not only the contact angle measured with water (*θ*_*w*_) but also the Lifshitz–van der Waals and acid-base interactions (electron donor and electron acceptor properties). To our knowledge, no previous work has discussed which hydrophobicity method could better explain the microbial adhesion behavior of *E. coli* strains.

To control biocontamination in food environments, it is important to understand the physicochemical aspects involved in the initial deposition of bacteria onto surfaces. Therefore, this study investigates the relation between the adhesion behavior of 12 *E. coli* strains isolated from food and their surface hydrophobicities using qualitative (*θ*_*w*_) and quantitative (Δ*G*_*iwi*_) approaches.

## 2. Materials and Methods

### 2.1. Bacterial Strain Selection

Twelve *Escherichia coli* strains isolated from food and identified at the Laboratory of Microbiology and Hygiene of Food and Environment of the Pasteur Institute Morocco were used in this study. One hundred samples received for a month from different food sources (catering, food industries, hotels, and supermarkets) were analyzed. The procedures for isolation of *E. coli* from different samples given in this protocol follow the ISO-9308-1:2014 standards [25].

Among the examined samples, 12 out of 100 samples were tested positive for *E. coli* isolation. Positive samples showed typical pink colonies on MacConkey agar and showed characteristic green metallic sheen on EMB agar. Biochemical characters showed citrate utilization test negative, H2S production in TSI agar negative, indole production positive, urease activity negative, methyl red test positive, Voges–Proskauer negative, and lysine decarboxylase positive. The isolates were confirmed also using a biochemical test (Api 20E, BioMérieux, France).

Typing of *E. coli* isolates was performed in the Laboratory of Microbiology and Hygiene of Food and Environment of the Pasteur Institute Morocco according to Kauffmann and Das Kauffmann [26]. The *E. coli* strains were typed (13; 19; 38; 64; 76; 65; EI1; EI2; EI3; EI4; EM3; and EM4) as a function of the samples' codes and then stored at −20°C in glycerol stocks before analysis.

### 2.2. Bacterial Growth Conditions and Preparation of Bacterial Suspensions

Each bacterial strain was incubated overnight at 37°C in Liquid Luria-Bertani (LLB) medium, which contains tryptone, yeast extract, and NaCl. After 24 h of incubation, bacterial cells were harvested by centrifugation at 8400 × g for 15 min, and washed twice with, and resuspended in, KNO_3_ solution with ionic strength 0.1 M. The bacterial optical densities (ODs) were adjusted using a spectrophotometer to approximately 0.7 and 0.8 corresponding to 10^8^ CFU/mL for subsequent experiments [9]. The bacterial suspension was filtered using a 0.45 *μ*m cellulose acetate filter (Sartorius). A thick lawn of cells was obtained after filtration by means of negative pressure. The wet filters were placed carefully on a glass support with double-sided sticky tape and allowed to air-dry until the so-called stable plateau contact angles could be measured [9].

### 2.3. Cleaning and Preparation of Solid Surfaces

The substrate used for the adhesion experiments was glass. The glass samples were microscope slides (RS, France). Before each experiment, substrates were immersed in 95% ethanol for 15 min and then rinsed six times with distilled water. Finally, the substrate was autoclaved for 15 min at 120^◦^C [9].

### 2.4. Cell Surface and Substratum Characterization

The physicochemical properties of the bacterial surface and substratum were determined using a goniometer (GBX instruments) by the sessile drop method according to Busscher [27]. The surface energy of bacteria and substratum surfaces was determined by measuring the contact angle with three liquids: water, formamide 99%, and diiodomethane 99%. Three to six drops of liquid were placed on each solid material and bacterial filter (described earlier).

The qualitative hydrophobicity expressed as *θ*_*w*_ was directly analyzed by measuring the contact angle with water. The quantitative hydrophobicity was determined using the Van Oss approach [24], which explains hydrophobicity as the free energy of interaction between two materials when immersed in water, denoted as ∆*G*_*iwi*_. The surface is considered hydrophobic or hydrophilic if its free energy is negative (∆*G*_*iwi*_ < 0) or positive (∆*G*_*iwi*_ > 0), respectively. The free energy can be estimated from the surface tensions of interacting entities according to the following formula:

(1) $$\begin{array}{c} \Delta G_{iwi}=-2\gamma_{iw}=-2\left({\left({\left(\gamma_{i}^{\text{LW}}\right)}^{1/2}-{\left(\gamma_{w}^{\text{LW}}\right)}^{1/2}\right)}^{2}+2\left({\left(\gamma_{i}^{+}\gamma_{i}^{-}\right)}^{1/2}+{\left(\gamma_{w}^{-}\gamma_{w}^{+}\right)}^{1/2}-{\left(\gamma_{i}^{+}\gamma_{w}^{-}\right)}^{1/2}-{\left(\gamma_{w}^{+}\gamma_{i}^{-}\right)}^{1/2}\right)\right). \end{array}$$

Furthermore, the electron donor (*γ*^−^) and acceptor (*γ*^+^) characteristics and the Lifshitz–van der Waals (*γ*^LW^) interactions were estimated by the approach proposed by Van Oss [24]; the contact angle can be expressed as follows:

(2) $$\begin{array}{c} \text{Cos}\theta =\frac{-1+2{\left(\gamma_{S}^{\text{LW}}\gamma_{L}^{\text{LW}}\right)}^{1/2}/\gamma_{L}+2{\left(\gamma_{S}^{+}\gamma_{L}^{-}\right)}^{1/2}/\gamma_{L}+2{\left(\gamma_{S}^{-}\gamma_{L}^{+}\right)}^{1/2}}{\gamma_{L}}, \end{array}$$

where *S* and *L* denote solid surface and liquid phases, respectively.

Lastly, the Lewis acid–base components can be identified as follows:

(3) $$\begin{array}{c} \gamma^{AB}=2{\left(\gamma_{S}^{-}\gamma_{S}^{+}\right)}^{1/2}. \end{array}$$

The surface free energy components of water, formamide, and diiodomethane are known (Table 1), but the corresponding values for the entity *i* have to be determined.

### 2.5. Adhesion Assay

The bacterial suspension was placed in a Petri dish containing the sterilized materials (glass). After 3 h of incubation at 25°C, nonadherent cells were eliminated by three consecutive rinses with sterile distilled water [28, 29]. The glass samples were dried at room temperature, and then a Gram coloration was performed to assess bacterial adhesion. The adhesion on the glass surface was examined by using an optical microscope (× 400) (Olympus CH30). To estimate the percentage of the surface occupied by adherent cells, the obtained images after observation by microscopy were treated using an algorithm developed in the MATLAB software program [8].

### 2.6. Statistical Analysis

All physicochemical analyses and adhesion assays were conducted with three repetitions. Three to six contact angle measurements were performed on each bacterial filter and glass surface. The correlations studies were carried out between water contact angle measurement (*θ*_*w*_), the free energy of interaction (Δ*G*_*iwi*_), and the percentage of surface occupied by adhered cells using SPSS 20.0 software.

## 3. Results

### 3.1. Cell Surface and Substratum Characterization

The physicochemical characteristics of both microbial cells and material surfaces play an essential role in microbial adhesion phenomena. To control and inhibit adhesion, an understanding of the mechanisms involved in the interaction between microbial cells and substrates is required. Thereby, the physicochemical surface properties of *E. coli* strains and glass surfaces were measured by the contact angle (Table 2).

Hydrophobicity is often expressed in terms of contact angle formed by a sessile drop of water *θ*_*w*_. In the present work, we used this parameter to express the qualitative hydrophobicity of both bacterial strains and substratum. According to Vogler [30], hydrophobic surfaces exhibit water contact angle values higher than 65°, whereas hydrophilic surfaces gave water contact angle values lower than 65°.

Table 2 illustrates the bacterial physicochemical characterization by the contact angle method. Based on contact angle with the water value *θ*_*w*_, the results show that *E. coli* strains codified EI1, 38, EM3, 64, 19, EM4, and EI4 showed hydrophobic values ranging from 69.44° to 99.8°; however, the *E. coli* strains 13, 76, EI3, EI2, and 65 were hydrophilic (33.22–56.5°).

Using the thermodynamic approach of Van Oss [20, 21, 24], the absolute degree of hydrophobicity was determined with Δ*G*_*iwi*_ equations expressed in formula (equation (1)). Based on this quantitative approach, the results in Table 2 show that *E. coli* strains codified EM4,13, 76, EI3, EI2, 65, and EI4 were also hydrophilic (*ΔG*_*iwi*_ > 0), whereas the *E. coli* strains EI1, 38, EM3, 64, and 19 were hydrophobic (Δ*G*_*iwi*_ < 0). The free energy of interaction Δ*G*_*iwi*_ ranged from hydrophilic (48.6 mJ/m^2^) to hydrophobic (−70.7 mJ/m^2^) values.

Referring to the results in Table 2, it appeared that the *E. coli* strains 13, 76, EI3, EI2, and 65 were hydrophilic according to both qualitative and quantitative approaches. Moreover, the glass surface was qualitatively (*θ*_*w*_ = 36.14°) and quantitatively (Δ*G*_*iwi*_ = 38.61 mJ/m^2^) hydrophilic.

### 3.2. Adhesion Tests

**Figure 1:**
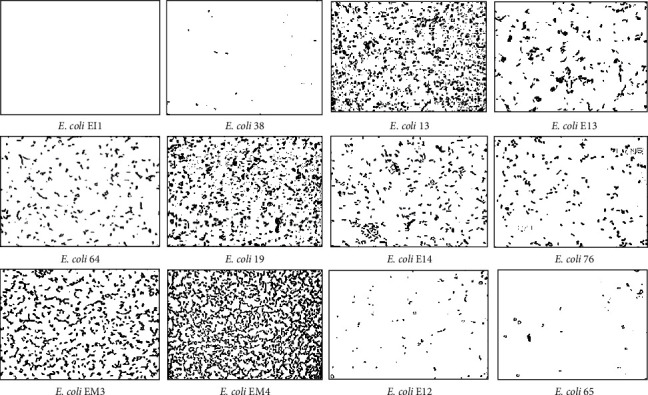
Microscopic images of *E. coli* strains adhesion to glass surface at × 400 magnification. Black spots are bacterial adhesion on glass surface.

**Figure 2:**
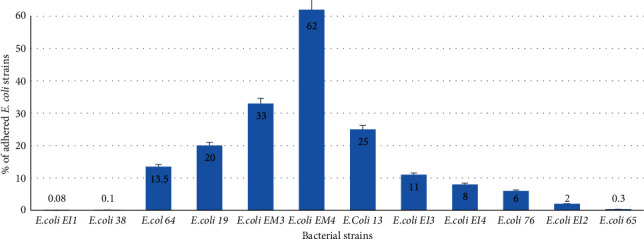
Percentages of the adhered cells of *E. coli* strains on glass substratum. Error bars represent standard deviations of the percentages of the occupied surface by bacteria. Adhesion assays were conducted with three repetitions.

The adhesion ability of *E. coli* strains isolated from food origins on an inert surface (glass) was examined. Optical microscopic images of *E. coli* strains after 3 h of contact on the glass are provided in Figure 1. The percentages of the occupied surface by cells on the glass are presented in Figure 2.

As observed in Figures 1 and 2, the adhesion ability of *E. coli* strains on a glass support varied among strains. In fact, *E. coli* strains 64, 19, EM3, EM4, 13, and EI3 showed a high capacity to adhere to the glass material (percentage of adhered cells 13.5%, 20%, 33%, 62%, 25%, and 11%, respectively). However, *E. coli* strains EI1, 38, EI4, 76, EI2, and 65 did not adhere significantly to the glass substratum (0.08%, 0.10%, 8%, 6%, 2%, and 0.3%, respectively). Furthermore, *E. coli* EM4 showed a strong adhesion capacity onto glass surface samples (62%).

A comprehensive understanding of *E. coli* adhesion relies on establishing how the physicochemical properties of bacterial surfaces are involved. Therefore, we investigated the correlation between the adhesion behavior of *E. coli* strains and their surface hydrophobicities with qualitative (*θ*_*w*_) and quantitative (Δ*G*_*iwi*_) approaches.

Using SPSS software, we analyzed several correlation models (linear, logarithmic, and exponential) between the percentage of adhered cells of *E. coli* strains and their surface hydrophobicities with both approaches (*θ*_*w*_; Δ*G*_*iwi*_) to identify the best correlation model revealing the relationship between the adhesion capacity of our strains and their surface properties.

**Figure 3:**
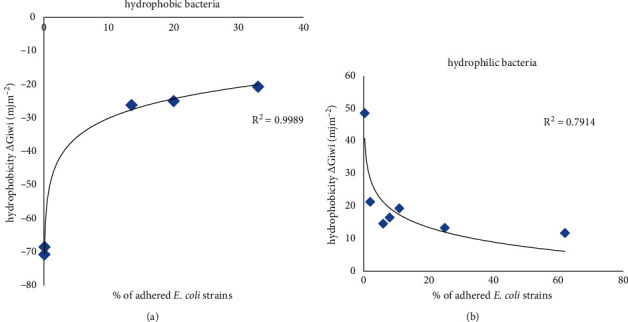
Logarithmic correlation between the bacterial surface hydrophobicity Δ*G*_*iwi*_ and the percentage of adhered cells of *E. coli* strains. (a) Hydrophobic bacteria ∆*G*_*iwi*_ < 0. (b) Hydrophilic bacteria ∆*G*_*iwi*_ > 0.

Correlation analysis showed no significant correlation between the cells' qualitative hydrophobicity (*θ*_*w*_) and their adhesion intensity; however, correlation results revealed a highly significant logarithmic relation between the adhesion behavior of our strains and their quantitative hydrophobicity Δ*G*_*iwi*_ (Figure 3). The coefficient of determination *R*^2^ indicates a good correlation between the percentage of adhered cells of *E. coli* and their surface hydrophobicity for hydrophobic (*R*^2^ = 0.9989) and hydrophilic (*R*^2^ = 0.7914) characteristics separately.

**Figure 4:**
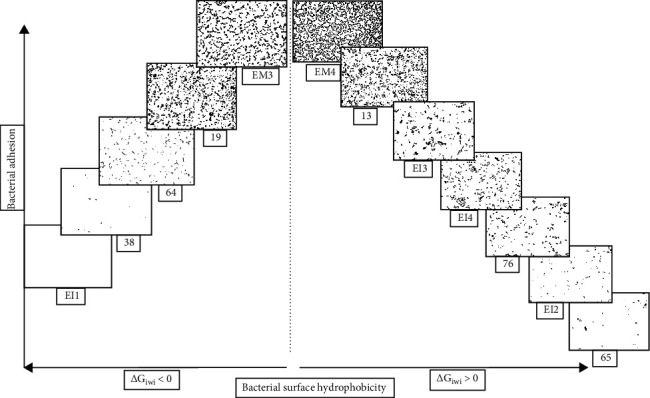
Schematic of *E. coli* strains adhesion behavior on glass according to their surface hydrophobicity (Δ*G*_*i*wi_).

For a better understanding of this relationship between the adhesion behavior of *E. coli* strains and their hydrophobic nature, Figure 4 illustrates the distribution of the percentage of *E. coli* adhered cells as a function of their quantitative hydrophobicity (Δ*G*_*iwi*_). The results indicated an increase and decrease in the percentage of adhered cells with negative (Δ*G*_*iwi*_ < 0) and positive (Δ*G*_*iwi*_ > 0) free energy of interaction values, respectively. In addition, the adhesion of *E. coli* strains onto the glass substrate was more pronounced when going from extreme negative and positive values of quantitative hydrophobicity (Δ*G*_*iwi*_) to centric values (close to 0); however, this behavior was not observed with qualitative hydrophobicity (*θ*_*w*_) (Figure 4).

## 4. Discussion

The adhesion of microorganisms to surfaces is the first step in biofilm formation. This adhesion stage is largely governed by physicochemical interactions, primarily Lifshitz–van der Waals, Lewis acid-base, and electrostatic ones. These interactions depend on the physicochemical properties that include hydrophobicity, electrostatic charge, and electron donor/electron acceptor characteristics.

Surface hydrophobicity is generally thought to be the main factor in microbial adhesion. Several authors consider hydrophobicity to be the key parameter governing bacterial adhesion to surfaces [4, 15, 31–36]. Contact angle with water expressed as wettability (*θ*_*w*_) is generally used to assess surface hydrophobicity. However, with this approach, it is only possible to evaluate hydrophobicity qualitatively [9, 10]. Therefore, Van Oss and coworkers [24] proposed a quantitative determination of surface hydrophobicity Δ*G*_*iwi*_ that takes into account several surface properties' parameters (the electron donor (*γ*−) and acceptor (*γ*^+^) characteristics and the Lifshitz–van der Waals (*γ*^LW^)).

Several research works have used either qualitative or quantitative hydrophobicity to explain the adhesive behavior of the studied microorganisms. However, to our knowledge, no previous studies have investigated which of these hydrophobicities' parameters could explain better the adhesive behavior of *E. coli* strains. Therefore, the present study aimed to evaluate the adhesion ability of 12 *E. coli* strains isolated from food to adhere to an inert surface (glass) and then investigate the relation between their surface hydrophobicities and their adhesion behavior according to quantitative (Δ*G*_*iwi*_) and qualitative (*θ*_*w*_) approaches.

Table 2 provides the contact angle measurement results and shows that the glass support had a hydrophilic surface as determined by both qualitative (*θ*_*w*_) and quantitative (Δ*G*_*iwi*_) approaches, which concurs with previous researchers [4, 13, 32, 35, 36]. The findings in Table 2 also indicate that *E. coli* strains had different hydrophobicities, depending on the two aforementioned approaches. In fact, only *E. coli* strains 13, 76, EI3, EI2, and 65 were hydrophilic according to these approaches. These results showed similarities with those of Hamadi [9, 12], who reported that *E. coli* strains isolated from patients with urinary tract infections are hydrophilic with both approaches.

According to the literature, the hydrophobicity of a bacterial cell is largely influenced by the residues and structures on the cell surfaces, which can be hydrophilic or hydrophobic [10, 19, 37]. Hence, bacterial hydrophobicity varies among species and strains, even within the same strain, depending on the mode and stage of growth, growth medium composition, and even the analysis technique [7, 9, 38, 39], potentially explaining the variation of hydrophilicities even within the same species in our work.

The adhesion assay indicated that *E. coli* strains showed a different ability to adhere to glass materials (Figure 1). To investigate the relation between the adhesion behavior of *E. coli* strains and their physicochemical surface properties, we evaluated the correlation between *E. coli* adhesion behavior and their surface hydrophobicities using qualitative (*θ*_*w*_) and quantitative (Δ*G*_*iwi*_) strategies. The results in Figure 3 demonstrated a strong logarithmic correlation between the free energy of the interaction (∆*G*_*iwi*_) and the adhesion of *E. coli* strains onto glass supports; however, no significant correlation with the qualitative hydrophobicity was observed. Similarly, previous studies investigating the relationship between the hydrophobicity properties (*θ*_*w*_) of *E. coli* and their attachment ability have not found a correlation between the degree of hydrophobicity of the supporting surfaces and the number of adhered cells [34, 40, 41].

Several works, including this one, have reported that qualitative hydrophobicity (*θ*_*w*_) is a general concept that cannot explain systematically the microbial adhesion results for many supports [4, 42, 43]. According to Goulter [2], qualitative hydrophobicity is a general concept that cannot be directly measured but it can be only estimated by observing the bulk properties of numerous cells and interpreting these interactions as those of molecules. This could be a primary factor that makes many studies unable to find a significant correlation between qualitative hydrophobicity and adhesion behavior [4, 7, 23].

Few studies have used the thermodynamic approach in the interpretation of adhesion behavior. However, this strategy could represent an important tool in understanding microbial adhesion because it takes into account the Lifshitz–van der Waals interactions and acid-base interactions that are known to play an essential role in adhesion [9, 44]. Based on the results obtained here, we suggest that the adhesion of *E. coli* strains on a glass substratum is mainly governed by surface quantitative hydrophobicity (Δ*G*_*iwi*_). Therefore, we propose that the free energy of interaction (Δ*G*_*iwi*_) should be further investigated as an important parameter to understand and predict the adhesion behavior of bacterial strains.

## 5. Conclusion

The mechanism of the initial attachment of *E. coli* strains to surfaces is most likely a complex process involving several factors. Many aspects, such as hydrophobicity, have been shown to play an essential role in cell attachment. This study demonstrated that qualitative hydrophobicity (*θ*_*w*_) could not explain systematically the adhesion behavior of 12 *E. coli* strains; however, the quantitative hydrophobicity (Δ*G*_*iwi*_) showed a good logarithmic relation with the percentage of adhered cells. This work provides an important approach for understanding the mechanisms underlying different aspects of adhesion behavior by focusing on studying the thermodynamic approach (Δ*G*_*iwi*_) as an important model of hydrophobicity that could predict *E. coli* adhesion behavior.

To end, a comparison of *E. coli* strains adhesion on other substrates and the study of their biofilm formation could better inform on their behavior according to their surface qualitative and quantitative hydrophobicities. This strategy could help in understanding and controlling the adhesive behavior of these strains in order to reduce biocontamination risks in food industries

## 6. Disclosure

This work was part of the Research Project, RS/2011/32: “Study of Biofilm Formation on Materials Used in Food Industry.” The project was part of the “Programme National du Developpement de la Recherche Sectorielle” launched by the National Centre for Scientific and Technical Research (CNRST) in collaboration with the Faculty of Science and Technology of Beni Mellal, Faculty of Science and Technology of Fez and Institute Pasteur, Morocco.

## Figures and Tables

**Table 1:** Surface energy of contact angle liquids according to van Oss [21].

Liquids	*γ* ^lw^ (mJ/m^2^)	*γ* ^+^ (mJ/m^2^)	*γ* ^−^ (mJ/m^2^)
Water	21.8	25.5	25.5
Formamide	39.0	2.3	39.6
Diiodomethane	50.8	0	0

*γ* ^LW^: Lifshitz–van der Waals forces; *γ*^−^: electron donor character; *γ*^+^: electron acceptor character.

**Table 2:** Contact angles, surface tension parameters, and free energy of interaction of *E. coli* strains and glass surface.

Strains and substratum	Contact angles (°)			Surface tension: components and parameters (mJm^−2^)			Free energy of interaction (mJm^−2^)
	*θ*_water_	*θ*_formamide_	*θ*_diiodomethane_	*γ* ^lw^	*γ* ^+^	Γ^−^	Hydrophobicity (Δ*G*_*iwi*_)
*E. coli* EI1	99.80 (1.23)	78.28 (3.2)	46.12 (4.02)	36.31	0.51	1.40	−70.7
*E. coli* 38	93.70 (0.67)	70.88 (0.34)	35.02 (3.22)	41.92	0.44	2.31	−68.5
*E. coli* EM3	77.58 (1.56)	66.74 (1.78)	53.7 (2.03)	32.09	0.25	13.84	−26.1
*E. coli* 64	74.03 (3.23)	63.35 (2.89)	46.85 (2)	35.92	0.13	15.27	−25.0
*E. coli* 19	74.2 (3.45)	63.85 (4.62)	64.61 (1.45)	25.87	0.60	14.93	−20.7
*E. coli* EM4	69.44 (2.38)	84.28 (2.01)	42.2 (3.66)	38.39	7.09	45.82	11.8
*E. coli* 13	54.15 (1.43)	53.6 (1.23)	45.41(0.96)	36.70	0.04	35.32	13.4
*E. coli* 76	56.5 (3.02)	57.8 (2.05)	64.92 (2.2)	25.69	0.62	35.15	14.6
*E. coli* EI4	75.38 (3.56)	92.06 (4.01)	64.2 (4.33)	26.11	4.84	42.71	16.6
*E. coli* EI3	33.22 (0.96)	34.58 (5.1)	73.42 (1.06)	20.95	5.15	46.11	19.3
*E. coli* EI2	42.52 (2.77)	44.22 (2.33)	65.68 (4.83)	25.28	1.98	42.67	21.3
*E. coli* 65	51.43 (1.43)	74.38 (0.89)	64.98 (1.36)	25.66	1.31	66.91	48.6
Glass	36.14 (3.2)	46.28 (1.53)	59.52 (2.15)	28.79	0.73	54.90	38.61

Standard deviation is given in the parentheses. *θ*_water_: contact angle with water/qualitative hydrophobicity; *θ*_formamide_: contact angle with formamide; *θ*_diiodomethane_: contact angle with diiodomethane; *γ*^LW^: Lifshitz–van der Waals forces; *γ*^−^: electron donor character; *γ*^+^: electron acceptor character; Δ*G*_*iwi*_: free energy of interaction/quantitative hydrophobicity.

## Data Availability

The data used to support the findings of this study are included within the article.
